# Transmission of influenza virus in temperate zones is predominantly by aerosol, in the tropics by contact: A hypothesis

**DOI:** 10.1371/currents.RRN1002

**Published:** 2009-08-17

**Authors:** Anice Lowen, Peter Palese

**Affiliations:** ^*^Mount Sinai School of Medicine and ^†^Mount Sinai School of Medicine, NY

## Abstract

Using the guinea pig model, we have previously shown that the aerosol transmission of a seasonal human influenza virus is blocked by humid (80% relative humidity) or warm (30°C) ambient conditions. In contrast, we found that transmission by a contact route proceeded at high efficiency despite increased temperature or humidity. Based on these findings, and the observed seasonal behavior of influenza viruses in various regions of the world, we hypothesize herein that the predominant mode of influenza virus transmission differs in temperate and tropical climates. Specifically, we predict that aerosol transmission predominates during the winter season in temperate regions, while contact is the major mode of spread in the tropics. With this idea in mind, possible explanations for the current summer-time spread of swine-origin influenza viruses are discussed.

## Modes of influenza virus transmission

Despite its fundamental importance to controlling the spread of infection, the mode with which influenza viruses transmit from host-to-host remains a point of significant controversy. Three vehicles of viral spread have been implicated: fomites, large respiratory droplets (usually said to be >10 μM in diameter), and small airborne droplet nuclei (usually said to be < 5 μM in diameter) [1]. Evidence supporting a role for each mode in the transmission of influenza viruses between humans has been reported [reviewed in [1] [2]] and under experimental conditions using the guinea pig model all three modes have been observed, albeit with differing efficiencies [3]. Based on the literature and our own findings, we propose that the possible modes of influenza virus transmission encompass the entire spectrum, from direct contact through to long-range airborne transmission. Which mode of transmission is the most important, or occurs with the highest frequency, is subject to the prevailing circumstances. These circumstances would include the types of surfaces available for fomite transmission, the rate and direction of air exchange, amount of virus shed from the infectious individual, the strain of influenza virus and – importantly – environmental conditions. The critical question then becomes, not what is the predominant mode of influenza virus transmission, but rather what conditions are favorable to each type of spread? The answer to this question would allow investigation of how variation in mode of transmission with prevailing conditions impacts the epidemiology of influenza. One aspect of this bigger picture in which we are particularly interested is the interplay between the mode of transmission and the seasonality of influenza.

### Influenza seasonality

In temperate regions of the world influenza epidemics occur with a marked winter-time seasonality. Thus, in the Northern Hemisphere, peak influenza activity is seen between November and March, while in the Southern Hemisphere, cases of influenza are most frequent from May to September (**Fig. 1**).

**Figure:**
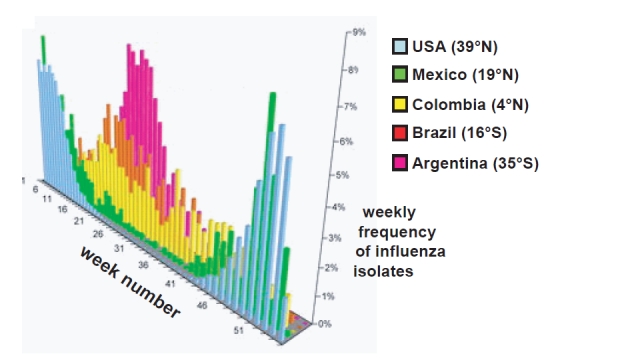
**Figure 1.** Weekly influenza activity for five countries spanning the Americas. Adapted from reference [4], this graph illustrates the differing patterns of influenza activity seen in temperate and tropical (here exemplified by Colombia) regions. The latitude of the capital city of each country is indicated in brackets in the figure legend.

In the tropics, by contrast, influenza activity tends to be much more sporadic, with isolations occurring either throughout the year or in temporally irregular outbreaks (Fig. 1) [4]. For many years the underlying reasons for influenza seasonality were widely discussed but poorly understood [5] [6]; recently, however, we have reported compelling evidence obtained in the guinea pig model which suggests that seasonal variation in humidity and temperature may direct seasonal fluctuations in influenza activity [7] [8]. In addition, as demonstrated below, our data revealed that transmission by the aerosol route (here defined as encompassing both large and small droplet spread) is acutely sensitive to both humidity and temperature, while transmission by the contact route is not.

### Sensitivity of aerosol transmission to humidity and temperature

By combining the guinea pig transmission model with the use of an environmentally controlled chamber, we have been able to systematically evaluate the effects of temperature and relative humidity on influenza virus transmission [7] [8]. To specifically test the effects on transmission by the aerosol route, we used an experimental design in which infected and exposed guinea pigs were placed in separate cages [7]. Each cage was open to air flow on the top and on one side and pairs of cages were placed at a minimum distance of approximately 2 cm such that the guinea pigs were not able to touch each other. Following this arrangement, four infected and four naïve animals were placed together into an environmental chamber and monitored for virus shedding over a period of one week. This strategy revealed a strong impact of ambient temperature and humidity conditions on the aerosol transmission of influenza viruses (**Fig. 2**). In general, we found that cold and dry conditions were most favorable, while aerosol transmission was completely blocked by warm (30°C) or humid (80% relative humidity) conditions.

**Figure:**
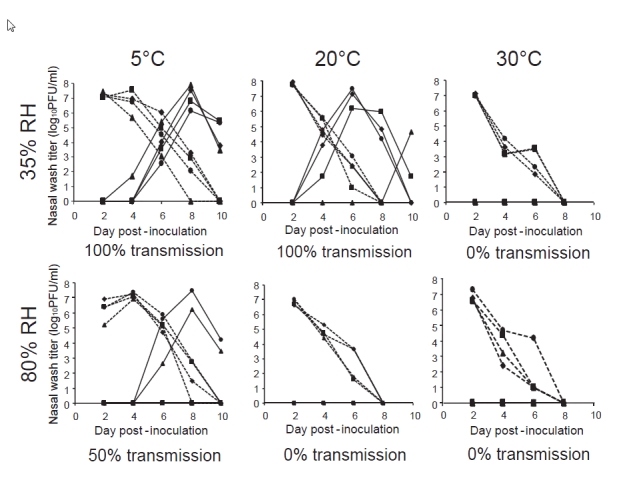
**Figure 2.** Aerosol transmission of influenza A/Panama/2007/1999 virus varies with relative humidity and temperature. Nasal wash titers of intranasally inoculated animals are indicated by dashed lines, while nasal wash titers of exposed animals are shown with solid lines. In each experiment, n = 4 inoculated guinea pigs and 4 exposed guinea pigs. The environmental conditions under which each experiment was performed are indicated at the top (temperature) and at the left (relative humidity, RH). Comparison of results across each row shows the decrease in aerosol transmission with increasing temperature; comparison of results down each column indicates the decrease in aerosol transmission with the increase in relative humidity [7].

### Insensitivity of contact transmission to humidity and temperature

To evaluate whether transmission by the contact route would also be affected by humidity and temperature, we used an experimental design in which infected and exposed guinea pigs were placed in the same cage together [8]. This set up allows for transmission to occur by direct or indirect contact, as well as by short-range aerosol transmission. As with the aerosol transmission experiments, environmental conditions were controlled within a chamber and exposure occurred over a period of one week. As shown in **Figure 3**, these experiments indicated that the efficiency of transmission by the contact route varies very little with relative humidity or temperature.

**Figure:**
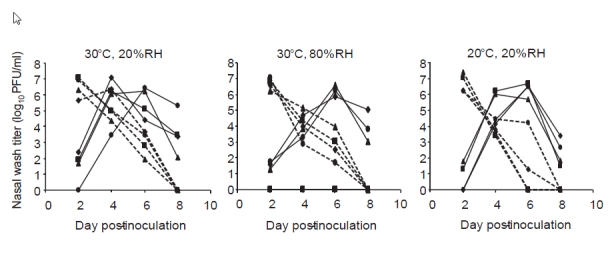
**Figure 3.** Contact transmission of A/Panama/2007/1999 virus is relatively insensitive to effects of temperature and humidity.  Nasal wash titers of intranasally inoculated animals are indicated by dashed lines, while nasal wash titers of exposed animals are shown with solid lines. In each experiment, n = 4 inoculated guinea pigs and 4 exposed guinea pigs. The environmental conditions under which each experiment was performed are indicated above the corresponding graph [8].

## Hypothesis: differing modes of transmission predominate in temperate and tropical zones

Overall, our data in the guinea pig model indicate that aerosol transmission is acutely sensitive to relative humidity and temperature, while contact transmission is not. An interesting hypothesis follows from these findings, namely that the transmission of influenza viruses during the winter season in temperate regions occurs predominantly by an aerosol route, while transmission year-round in the tropics is mainly through direct or indirect contact. If correct, this hypothesis could explain the documented differences in seasonality between temperate and tropical regions of the world. Specifically, if viral spread in the tropics relies mainly on fomites or direct contact, then transmission would be expected to proceed year-round despite hot and humid climatic conditions. The persistence of influenza viruses in the human populations of the tropics is likely of great importance to the global epidemiology of seasonal influenza: spread throughout the year in tropical regions is thought to allow the seeding of new influenza virus strains into the Northern and Southern Hemispheres in their respective autumn months [9]. Thus, the ability of influenza viruses to transmit by multiple different routes, which vary in their sensitivity to environmental conditions, may be essential to the maintenance of these viruses in the global human population.

### Summer-timer transmission of swine-origin H1N1 influenza viruses

In recent months, a very interesting exception to the standard seasonality of influenza has developed. Following its introduction into humans in the spring of 2009, the Northern Hemisphere has seen extensive spread of a swine-origin influenza virus (SOIV) over the spring and summer months [10]. Several possible explanations for this phenomenon exist: i) the increased number of summer cases is simply due to increased surveillance and testing of patients with influenza-like illness; ii) a unique viral trait allows SOIV strains to transmit by the aerosol route even under warm or humid conditions; iii) unlike conventional human strains, SOIV is able to transmit by aerosol under unfavorable conditions due to the lack of immunity against it in the human population; or iv) the transmission currently being seen in the Northern Hemisphere is occurring by the contact route and is occurring with unusually high efficiency due to the low level of immunity in the population. Given that the conventional H1N1 and H3N2 viruses which circulated in the winter of 2008/09 have not been detected out of season in high numbers, we feel that the first explanation is most likely incorrect. The remaining hypotheses are all feasible and time and experimentation will tell which is correct. At the present time, our favored hypothesis is the fourth, that summer-time transmission by a contact route is occurring with increased frequency due to the antigenic novelty of the strain. If correct, we would expect increased transmission in the coming winter months. In addition, if SOIV becomes established in the human population over the long term, then we would expect to see subsequent epidemics fall into the conventional pattern of winter-time seasonality.

## Acknowledgements

Figure 1 is derived from the published work of C. Viboud, W.J. Alonso and L. Simonsen.  

### Funding information

Funding for this work was provided by the Center for Research on Influenza Pathogenesis (NIAID contract HHSN266200700010C) and the W. M. Keck Foundation (to P.P.). A.C.L. is a Parker B. Francis Fellow in Pulmonary Research.

### Competing interests

The authors have declared that no competing interests exist.
